# A retrospective audit of fluid management in patients in an acute hospital and adherence to the GIFTASUP 2009 guidelines

**DOI:** 10.1186/1753-6561-9-S1-A22

**Published:** 2015-01-14

**Authors:** F Alhomida, J Hussain, F Macnamara

**Affiliations:** 1 Department of Surgery, Beaumont Hospital, Dublin 9. Ireland

## Background

Our audit entails the Intravenous fluids prescribed to postoperative patients by clinicians, predominantly the hospital-based interns, where they are analysed and compared with the GIFTASUP 2009 guidelines on prescribing. We gathered data on the attitudes and competency levels in prescribing of the interns, by directly surveying the intern population with questionnaires. We then outlined areas where IV fluid prescription is inconsistent with GIFTASUP and tried to address these inconsistencies with awareness and teaching sessions targeted at those most responsible for prescribing postoperative IV fluids. We then re-evaluated the prescription of IV fluids and measured change in prescription habits. Our audit did not measure morbidity and mortality associated with improper fluid prescription, rather the prescribing habits of clinicians.

## Methods

Data from 93 patient charts was collected and recorded in two phases, 50 patient charts before and 43 patient charts after an intern teaching session. Prior to the teaching session a questionnaire was handed out to 30 interns. The data was then analysed to see the level adherence patterns to the GIFTASUP guidelines across various surgical wards in Beaumont Hospital.

## Results

During the first evaluation only 14% of the patient charts were identified as correctly following the GIFTASUP guidelines, this increased to 30% during the second evaluation. Furthermore, based on our questionnaire 10% of interns were identified as not confident in their IV fluid prescribing skills. Additionally, 1 in 3 interns have not been taught about prescribing IV fluids in medical school, while only 20% of the interns who completed the questionnaire have been taught about prescribing IV fluids during their internship.

## Conclusions

Beaumont Hospital currently has no IV fluid guidelines set in place. This leaves many clinicians, especially interns with little access to the proper protocols in prescribing IV fluids. Further emphasis needs to be placed on the proper education and training of clinicians at the undergraduate and intern level.

